# Urinary Microbiome of Reproductive-Age Asymptomatic European Women

**DOI:** 10.1128/spectrum.01308-22

**Published:** 2022-11-16

**Authors:** Svetlana Ugarcina Perovic, Magdalena Ksiezarek, Joana Rocha, Elisabete Alves Cappelli, Márcia Sousa, Teresa Gonçalves Ribeiro, Filipa Grosso, Luísa Peixe

**Affiliations:** a UCIBIO–Applied Molecular Biosciences Unit, REQUIMTE, Faculty of Pharmacy, Department of Biological Sciences, Laboratory of Microbiology, University of Porto, Porto, Portugal; b Associate Laboratory i4HB, Institute for Health and Bioeconomy, Faculty of Pharmacy, University of Porto, Porto, Portugal; Wayne State University

**Keywords:** midstream urine, extended culturomics, 16S rRNA gene amplicon sequencing, *Lactobacillus*, *Gardnerella*, *Corynebacterium*

## Abstract

The knowledge of bacterial species diversity within the female urinary microbiome (FUM) is essential for understanding the role of the FUM in urinary tract health and disease. This study aimed to characterize the bacterial species diversity of the FUM of asymptomatic reproductive-age European women by combining extended culturomics and long-read sequencing of the near-full-length 16S rRNA gene. A total of 297 bacterial species (median of 53 species/sample) were identified, yet only 22% of the species were detected by both culture and sequencing methods. Recently recognized *Gardnerella*, *Lactobacillus*, and *Limosilactobacillus* species and 5 new putative *Corynebacterium* species were identified by culturomics, while anaerobic species (e.g., 11 *Peptoniphilus* spp.) were mostly detected by amplicon sequencing. Notably, there was not a single species common to all samples, although members of the genus *Lactobacillus* were detected in all. Lactobacillus crispatus, Lactobacillus iners, and Lactobacillus mulieris were observed in high relative abundance in several samples, as well as other species (e.g., Streptococcus agalactiae, Fannyhessea vaginae, Gardnerella vaginalis, Gardnerella swidsinskii), while low-abundance members (e.g., Finegoldia magna) were often more prevalent. A moderate correlation (Mantel test; *r* = 0.5) between community structure types captured by culturomics and amplicon sequencing was observed, highlighting the benefit of combining both methodologies. This study provided a detailed FUM structure at the species level, which is critical to unveil the potential relationship between specific microbiome members and urinary diseases/disorders. Moreover, the different capacity to characterize microbiome profiles of culturomic and amplicon sequencing is described, providing valuable insights for further urinary microbiome studies.

**IMPORTANCE** The bacterial species diversity within the female urinary microbiome (FUM) has been insufficiently characterized. This study demonstrated that complementarity between optimized culture-dependent and -independent approaches is highly beneficial for comprehensive FUM species profiling by detecting higher FUM species diversity than previously reported, including identification of unreported species belonging to the genera *Lactobacillus*, *Limosilactobacillus*, and *Latilactobacillus* and putative novel *Corynebacterium* species. Although some species were present in high relative abundance, low-abundance members were more prevalent. FUM classification into community structure types demonstrated high interindividual differences in urinary microbiome composition among asymptomatic women. We also report moderate correlation between culture-dependent and -independent derived data—highlighting drawbacks of each methodological approach. Our findings suggest that FUM bacterial diversity reported from previous studies may be underestimated. Finally, our results contribute to the fundamental knowledge of the FUM required for further exploration of the urinary microbiome role in urinary tract diseases.

## INTRODUCTION

Emerging studies of the female urinary microbiome (FUM) have suggested the importance of this unique bacterial community in maintaining urinary tract (UT) health (1–6). Advances in FUM characterization through next-generation sequencing and culture-based methodologies has allowed identification of FUM members and indication of their association with various UT conditions. These breakthrough findings have triggered the reassessment of current diagnosis practice for urinary tract infection (UTI) (7, 8) and the investigation of the role of the FUM in the poorly understood etiologies of UT disorders such as overactive bladder syndrome, urgency urinary incontinence, and interstitial cystitis/bladder pain syndrome (9–11).

To date, studies have described healthy FUM as a community dominated by certain genera, such as *Lactobacillus*, *Gardnerella*, or Streptococcus, or a mixed community without a single dominant genus involving, e.g., the combination of Staphylococcus, *Corynebacterium*, and *Prevotella* genera (10, 12–14). Although the composition of the healthy urinary microbiome of asymptomatic reproductive-age women at the genus level is relatively established, its species-level composition has not been comprehensively studied. Available studies point to the dominance of, e.g., Lactobacillus crispatus, Lactobacillus jensenii, and *Gardnerella* spp. (often mistakenly reported as Gardnerella vaginalis) (15) and the presence of certain potential uropathogens such as Escherichia coli and Enterococcus faecalis, usually observed in low amounts (7, 13, 16, 17). In fact, detailed species-level characterization is essential to understand FUM diversity and identify key functions contributing to urinary health and disease, since specific features are often species or even strain specific.

In more recent years, whole-genome shotgun metagenomic sequencing of the urinary microbiome (18–22) has enabled promising species-level profiling and functional potential exploration mostly of symptomatic and older human populations. Still, there are significant methodological challenges in obtaining high-quality sequencing data from low-biomass samples such as urine from asymptomatic individuals (23, 24) and preventing human genomic contamination (19).

Currently, the most commonly methodological approaches used for FUM characterization involve culturomics, an approach that relies on extensive sample culturing on several microbiological media under different conditions, allowing the recovery of a significantly higher number of bacteria that would not growth under routine conditions (3, 4), coupled with matrix-assisted laser desorption ionization–time of flight mass spectrometry (MALDI-TOF MS) as the primary identification method and/or DNA sequencing methodologies targeting individual short hyper-variable regions of the 16S rRNA gene (3, 9, 10, 12, 25). However, some methodological drawbacks complicate identification at the species level of some FUM members. For instance, culture-based methodologies with limited growth conditions and with insufficient resolution for identification of isolates do not fully capture bacterial species diversity, while short-read DNA-based methods are often limited to a reliable identification of FUM members only at the genus level (26). In fact, only a few studies combined cuturomics with large-scale whole-genome sequencing to comprehensively characterize FUM at the species and strain levels (18).

Using a comprehensive and accurate culturomics approach, we previously unveiled new *Lactobacillus* and *Limosilactobacillus* species and identified bacterial species involved in FUM shifts at two distant time points (17). These data highlight the existence of resilient FUM (e.g., composed of abundant L. crispatus) that maintained species-level composition over long periods of time but also the possibility of interchange between certain bacterial groups that might share common metabolic functions (e.g., a *Gardnerella swidsinskii*, *Fannyhessea vaginae*, and Dialister micraerophilus community type converted to a Gardnerella vaginalis, *Bifidobacterium* spp., and Cutibacterium avidum type).

In this study, to further improve the understanding of the FUM composition at the species level, we applied a combination of extended culturomics and 16S rRNA gene long-read sequencing.

## RESULTS

### Overview of the asymptomatic female study cohort.

Our study cohort comprised 20 female participants aged 24 to 38 years (average, 31; standard deviation, 4). Most women identified themselves as Portuguese nationality (80%), followed by other European nationalities (20%). Average body mass index was 21.9 kg/m^2^. Most women had a normal menstrual cycle (90%) and used contraceptives (85%), with few having experienced at least one pregnancy (25%). Characteristics of our study cohort comprising asymptomatic highly educated women included clinical and behavioral questionnaire data (personal medical history, UT health and infection history, pregnancy history, demographic and lifestyle information); results of urine dipstick and sediment microscopic analyses are available in Tables S3 and S4 in the supplemental material.

### Characterization of community structure types by culturomics.

Using extended culturomics, we observed a high bacterial load in urine samples (10^3^ to 10^8^ CFU/mL, ≥10^4^ CFU/mL in 80% of samples). A total of 2,043 isolates were studied (median, 103 isolates/sample) and assigned to 131 species (median, 20 species/sample) and 54 genera, as identified either by MALDI-TOF MS and/or sequencing of the most suitable genetic markers (Table S5). In this cohort, (we identified for the first time 13 bacterial species from different genera [Dermacoccus nishinomiyaensis, Gardnerella leopoldii, *Gardnerella swidsinskii*, *Gardnerella* genomospecies 3, Globicatella sulfidifaciens, *Lactobacillus mulieris*, Lactobacillus paragasseri, Limosilactobacillus urinaemulieris, Limosilactobacillus portuensis, Limosilactobacillus mucosae] [formerly Lactobacillus mucosae], Pseudoglutamicibacter cumminsii, Staphylococcus carnosus, and Staphylococcus equorum) and 5 putative novel *Corynebacterium* species (Table S5 and Fig. S1). Alpha diversity varied from 0.001 to 2.65 (median Shannon index [H′], 1.5). Bacterial species detected by culturomics and their relative abundance (RA) per sample are listed in Table S5. Of note, *Corynebacterium* (18 species), Staphylococcus (14 species), Streptococcus (10 species), *Lactobacillus* (7 species), and *Actinomyces* (6 species) were the genera that presented the highest species-level diversity.

**FIG 1:**
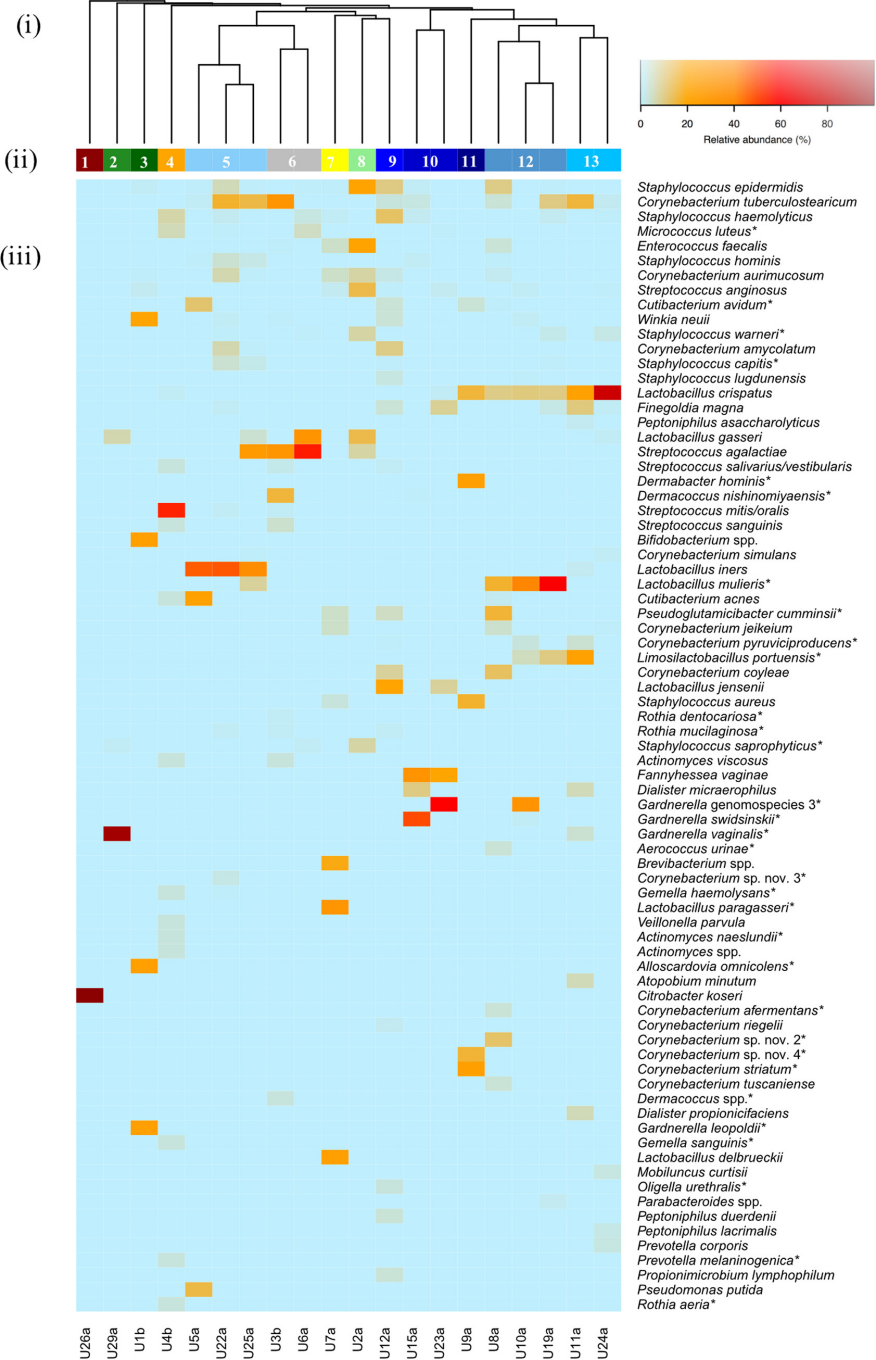
Species-level community structure types of the FUM by culturomics. (i) Hierarchical clustering of Bray-Curtis dissimilarity distance matrices on the relative proportions of CFU/mL within individual urine samples. (ii) Bars below the dendrogram denote community structure types. (iii) Heatmap of RA of bacterial species within each urinary microbiome. Only species that are at least 1% abundant in at least one sample are shown in order of decreasing prevalence (from top to bottom). Asterisks denote detection only by culturomics and not by amplicon sequencing.

Clustering the FUM into community structure types (CST) was performed at the genus and species levels (samples in the same CST shared >80% similarity by Bray-Curtis distance). Hierarchical clustering at the genus level identified 3 CST (Fig. S2). The most common CST was CST3 (*n* = 15/20), largely dominated by *Lactobacillus* in combination with other genera (e.g., Staphylococcus, *Corynebacterium*, Streptococcus, and *Cutibacterium*), followed by CST2 (*n* = 4), characterized mostly by *Gardnerella*, and CST1, dominated by *Citrobacter* (*n* = 1). On the other hand, species-level clustering resulted in 13 CST (Fig. 1, Table 1), mostly representing individual urine specimens, as only 5 CST included more than one sample. With the exception of 2 clusters dominated by a single bacterial species (CST1: Citrobacter koseri, CST2: Gardnerella vaginalis, >90%), the remaining CST were predominantly represented by a diverse bacterial community (different combinations and RA of bacterial species), which varied widely from 1.21 ± 0.05 to 2.65 as calculated by the Shannon diversity index (Fig. 1, Table 1). For instance, CST5 was characterized by combination of Lactobacillus iners with other bacterial species (e.g., Corynebacterium tuberculostearicum), CST12 included Lactobacillus crispatus, *Lactobacillus mulieris*, and other bacterial species, and CST10 comprised abundant *Fannyhessea vaginae* (formerly Atopobium vaginae), low-abundance Streptococcus anginosus, and in one sample, highly abundant *Gardnerella swidsinskii* (RA, ~50%) (Fig. 1).

**TABLE 1:** Overview of all community structure types and their characteristic species by culturomics^*a*^

Structure type	Characteristic species	Sample(s)	Shannon index (mean H′ ± SD)
1	Citrobacter koseri	U26a	0.001
2	Gardnerella vaginalis Lactobacillus gasseri	U29a	0.33
3	*Gardnerella leopoldii* Alloscardovia omnicolens *Bifidobacterium* spp. Winkia neuii Streptococcus anginosus	U1b	1.61
4	Streptococcus mitis*/*Streptococcus oralis Staphylococcus haemolyticus Micrococcus luteus *Actinomyces* spp. Lactobacillus crispatus	U4b	1.85
5	Lactobacillus iners Corynebacterium tuberculostearicum Staphylococcus epidermidis Staphylococcus hominis Staphylococcus capitis	U5a, U22a, U25a	1.61 ± 0.20
6	Streptococcus agalactiae Streptococcus salivarius */* Streptococcus vestibularis Micrococcus luteus Staphylococcus haemolyticus	U3b, U6a	1.40 ± 0.35
7	*Lactobacillus paragasseri* Lactobacillus delbrueckii *Brevibacterium* spp. Pseudoglutamicibacter cumminsii Corynebacterium jeikeium	U7a	1.87
8	Enterococcus faecalis Staphylococcus epidermidis Lactobacillus gasseri Streptococcus anginosus Corynebacterium aurimucosum	U2a	1.97
9	Lactobacillus jensenii Staphylococcus haemolyticus Staphylococcus epidermidis Corynebacterium amycolatum Corynebacterium coyleae	U12a	2.65
10	*Fannyhessea vaginae* Streptococcus anginosus	U15a, U23a	1.21 ± 0.05
11	Corynebacterium striatum Dermabacter hominis Staphylococcus aureus *Corynebacterium* sp. nov. 4 Lactobacillus crispatus	U9a	1.72
12	Lactobacillus crispatus *Lactobacillus mulieris* Staphylococcus epidermidis Cutibacterium avidum	U8a, U10a, U19a	1.77 ± 0.45
13	Lactobacillus crispatus Corynebacterium tuberculostearicum Finegoldia magna	U11a, U24a	1.52 ± 0.58

a Shared species within a structure type are presented in order of decreasing RA (RA, >1%, only top 5 shown).

### Characterization of community structure types by amplicon sequencing.

A total of 58,534 reads were generated, with most of them being assigned to the species level (88%; 51,317 reads). One sample (U6a) had <1,000 reads and was excluded from the analysis, while for the remaining, a median of 2,493 reads/sample (interquartile range [IQR], 1,625 to 3,920) were generated. A total of 231 species (IQR, 5 to 115; median, 39 species/sample) belonging to 107 genera and 8 phyla were identified. The alpha diversity varied from 0.135 to 2.79 (median H′, 0.90). Bacterial species detected by amplicon sequencing and their RA are listed in Table S6. Of note, *Corynebacterium* (16 species), *Peptoniphilus* (11 species), *Anaerococcus* (10 species), Streptococcus (9 species), and *Bacteroides* (8 species) were the genera that presented the highest species-level diversity.

**FIG 2:**
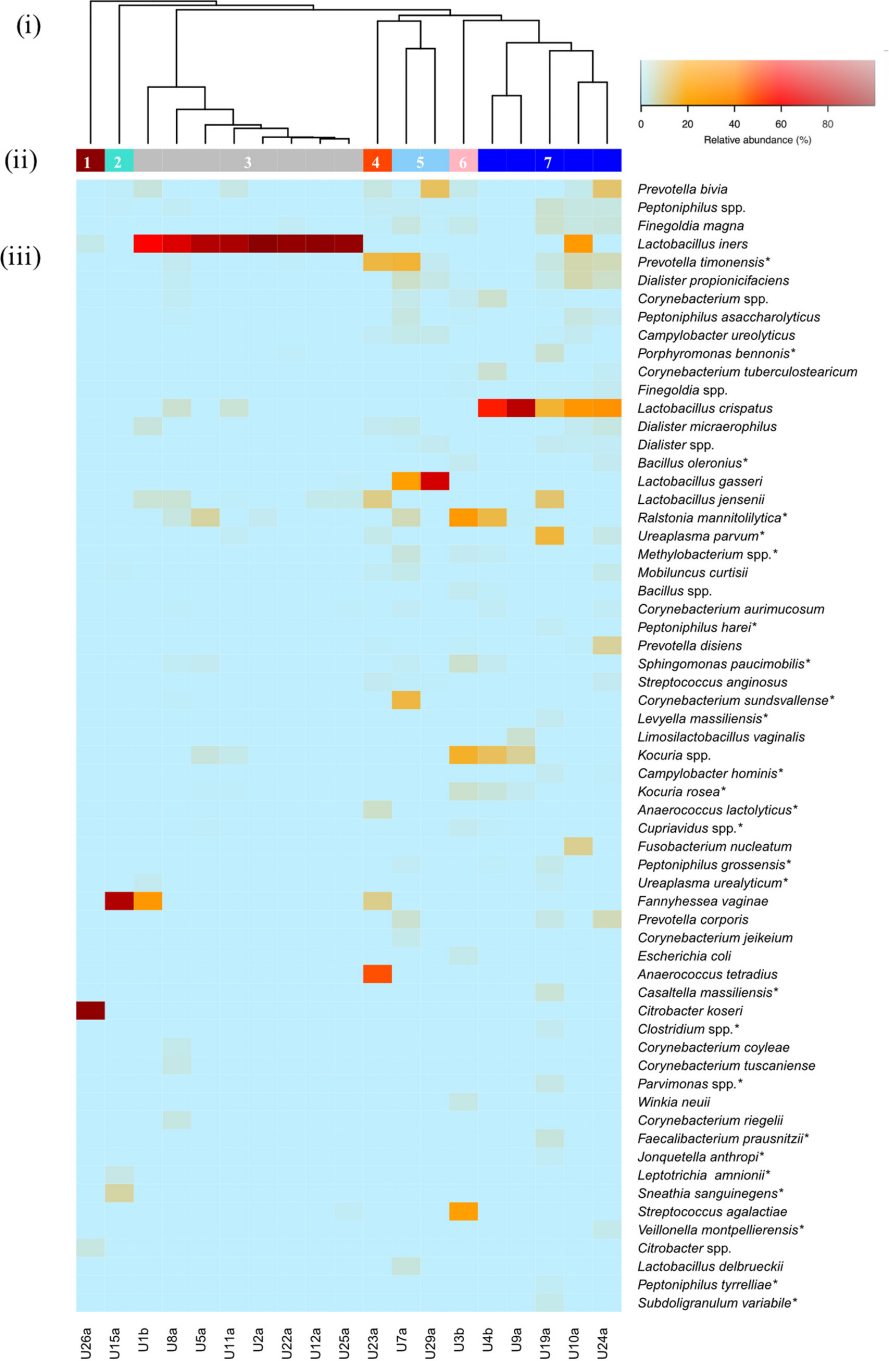
Species-level community structure types of the FUM by amplicon sequencing. (i) Hierarchical clustering of Bray-Curtis dissimilarity distance matrices on the relative proportions of reads for each OTU within individual urine samples. (ii) Bars below the dendrogram denote community structure types. (iii) Heatmap of RA of bacterial species within each urinary microbiome. Only species that are at least 1% abundant in at least one sample are shown in order of decreasing prevalence (from top to bottom). Asterisks denote detection only by amplicon sequencing and not by culturomics.

The same FUM clustering approach was applied to amplicon sequencing data. Genus-level clustering resulted in 5 CST (Fig. S3). The *Lactobacillus* genus in combination with other bacterial genera (e.g., *Prevotella*, *Dialister*, and *Corynebacterium*) represented the most prevalent CST (CST5; 79%, *n* = 15/19). Species-level clustering resulted in 7 CSTs (Fig. 2, Table 2), with the 3 (*n* = 15) most common being characterized by combination of a highly abundant *Lactobacillus* species (CST3: *L. iners*, CST5: Lactobacillus gasseri, CST7: L. crispatus) and species from other genera (Fig. 2). Remarkably, the Lactobacillus iners enriched CST was characterized by a reduced species diversity (CST3; H′, 0.56 ± 0.42) compared to other *Lactobacillus* CSTs. The remaining CSTs included highly abundant *C. koseri* (CST1; *n* = 1/19), *Fannyhessea vaginae* (CST2; *n* = 1/19), or a combination of different species (CST4: Anaerococcus tetradius and Prevotella timonensis, CST6: Ralstonia mannitolilytica and Streptococcus agalactiae; *n* = 1 each).

**TABLE 2:** Overview of all community structure types and their characteristic species by amplicon sequencing^*a*^

Structure type	Characteristic species	Sample(s)	Shannon index (mean H′ ± SD)
1	Citrobacter koseri *Citrobacter* spp. Lactobacillus iners	U26a	0.21
2	*Fannyhessea vaginae* Sneathia sanguinegens	U15a	0.59
3	Lactobacillus iners Prevotella timonensis	U1b, U8a, U5a, U11a, U2a, U22a, U12a, U25a	0.56 ± 0.42
4	Anaerococcus tetradius Prevotella timonensis Lactobacillus jensenii *Fannyhessea vaginae* Ureaplasma parvum	U23a	1.78
5	Lactobacillus gasseri Prevotella timonensis Dialister propionicifaciens Campylobacter ureolyticus	U7a, U29a	1.70 ± 1.13
6	Ralstonia mannitolilytica Streptococcus agalactiae *Kocuria* spp.	U3b	2.12
7	Lactobacillus crispatus *Corynebacterium* spp. Corynebacterium tuberculostearicum *Peptoniphilus* spp.	U4b, U9a, U19a, U10a, U24a	1.86 ± 0.85

a Shared species within a structure type are presented in order of decreasing RA (RA, >1%, only top 5 shown).

### Correlation between community structure types assigned by culturomics and amplicon sequencing.

A moderate correlation was observed using the Mantel test (*r* = 0.5, *P* < 0.05) between the CSTs assigned by culturomics and amplicon sequencing. Congruence was observed for the types of highly abundant *C. koseri* and combinations of different *Lactobacillus* species (Fig. 1 and 2), with 37% of samples (7/19) clustering into the same CST by both methodologies (Fig. S4).

**FIG 3:**
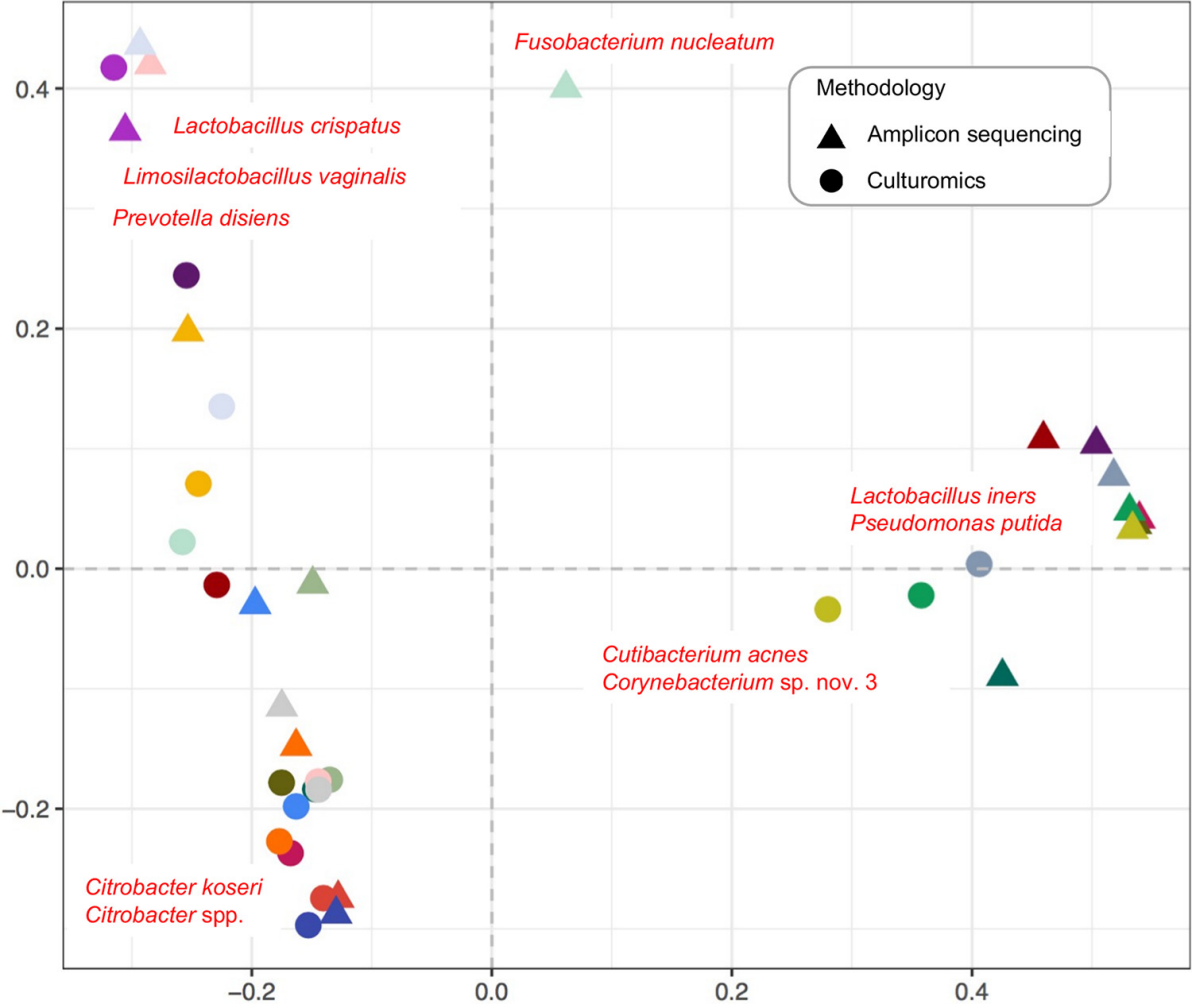
Biplot of the principal coordinate analysis (PCoA) based on the species-level Bray-Curtis dissimilarity matrices. Two-dimensional distances identify dissimilarities between bacterial community structures detected by culturomics and amplicon sequencing. The biplot, based on weighted average of the species scores, shows the top 10 species with the largest contributions to dissimilarities. Same colors indicate the same sample.

*Lactobacillus* species, among others, were responsible for the reduction in correlation between CSTs detected by different methodologies (e.g., Lactobacillus iners was more frequently detected in a higher RA by amplicon sequencing, while Cutibacterium acnes was more frequently detected by culturomics) (Fig. 3). Overall, amplicon sequencing enabled the detection of bacteria that are difficult to grow by conventional methods (e.g., Ureaplasma urealyticum, Ureaplasma parvum) and improved detection of fastidious bacterial species (e.g., Campylobacter ureolyticus, Finegoldia magna, *Fannyhessea vaginae*), whereas culturomics allowed the identification of various Gram-positive bacteria (e.g., Enterococcus faecalis, Streptococcus agalactiae, Streptococcus anginosus). Remarkably, some species detected by sequencing in low-read counts (e.g., Staphylococcus aureus and Actinomyces urogenitalis, RA, <0.1%) were identified by extended culturomics, confirming their presence in a given sample (Tables S5 and S6). Culturomics also allowed precise identification of closely related and/or newly described bacterial species, e.g., *Gardnerella leopoldii*, *Gardnerella* genomospecies 3, *Gardnerella swidsinskii*, Limosilactobacillus portuensis, *Limosilactobacillus urinaemulieris*, *Lactobacillus paragasseri*, *Lactobacillus mulieris*, and putative novel *Corynebacterium* species.

### Overview of bacterial species in the urinary microbiome of asymptomatic women.

In total, we captured an extended set of bacteria belonging to 8 phyla, 116 genera, and 297 species (median, 53 species/sample) in FUM of asymptomatic women (Tables S5 and S6, Fig. S5). Out of 297 species, we have identified 65 species (22% of total species) belonging to 35 genera and 5 phyla by both methodologies. Certain genera were characterized by outstandingly high species-level diversity that could be captured only by combined culture-based and DNA-dependent approaches. For instance, from a total of 25 *Corynebacterium* species, 8 could be identified by both methodologies (apart from 10 detected only by extended culturomics— including 5 putative novel species—and 7 only by amplicon sequencing), and from 14 species belonging to the *Lactobacillaceae* (4 genera; *Lactobacillus*, *Limosilactobacillus*, *Lacticaseibacillus*, and *Latilactobacillus*), 7 could be detected by both methodologies (in addition to 4 identified only by extended culturomics and 3 by amplicon sequencing) (Tables S5 and S6).

**FIG 4:**
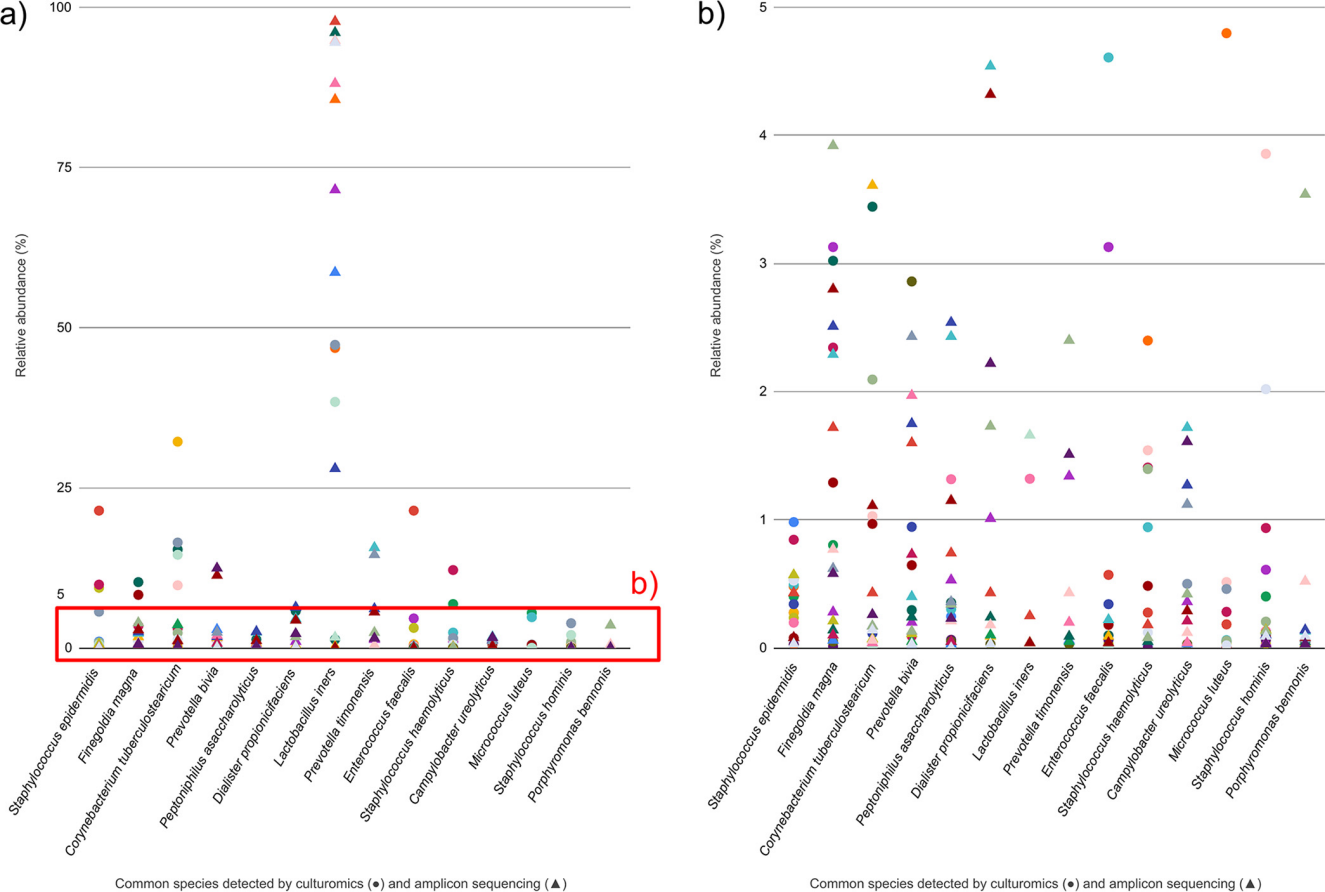
Common bacterial species of the FUM detected by culturomics and amplicon sequencing. (a) RA per sample of species present in more than 50% of samples by culturomics and amplicon sequencing. Only species that are detected by culturomics or amplicon sequencing with at least 1% abundance in at least one sample are presented in order of decreasing prevalence (from left to right). Same colors indicate the same sample. The red box indicates (b) close-up of section of panel a showing the RA range 0.01 to 5%.

We could not identify a single species present in all samples, although the genus *Lactobacillus* was detected in all. Instead, we were able to unveil 14 prevalent bacterial species (present in more than 50% of samples) with at least 1% of abundance in the sample (Fig. 4, Table S7). Staphylococcus epidermidis was the most common species (*n* = 18/20), followed by Finegoldia magna (*n* = 16/20), Corynebacterium tuberculostearicum (*n* = 15/20), and Prevotella bivia (*n* = 15/20) (Table S7). Remarkably, the common species were mostly low-abundance members (RA, <5%) (Fig. 4).

Additionally, we looked for the presence of opportunistic pathogens associated with the urogenital tract and found 16 bacterial species largely varying in their RAs (IRQ, 0.03 to 96.62%), among which Enterococcus faecalis, Streptococcus anginosus, and Ureaplasma parvum were the most frequently identified by both methodologies (Table 3). Notably, *C. koseri* was a highly abundant member detected by both methodologies, while *Fannyhessea vaginae* was only detected by amplicon sequencing. All opportunistic pathogens associated with the urogenital tract detected by culturomics and/or amplicon sequencing are listed in Table 3.

**TABLE 3:** Opportunistic pathogens associated with the urogenital tract^*a*^

Species	Frequency in FUM^*b*^ (%)	Culturomics		Amplicon sequencing	
		Frequency in 20 samples (%)	RA (%)	Frequency in 19 samples (%)	RA (%)
Enterococcus faecalis	12/20 (60%)	11/20 (55%)	0.01–20	3/19 (16%)	0.04–0.22
Streptococcus anginosus	11/20 (55%)	10/20 (50%)	0.06–13.33	7/19 (37%)	0.03–1.31
Ureaplasma parvum	8/20 (40%)	ND	ND	8/19 (42%)	0.10–15.10
Escherichia coli	6/20 (30%)	4/20 (20%)	0.02–0.28	4/19 (21%)	0.04–1.54
Streptococcus agalactiae	6/20 (30%)	6/20 (30%)	0.03–55.96	2/19 (10%)	0.85–23.20
Ureaplasma urealyticum	5/20 (25%)	ND	ND	5/19 (26%)	0.08–1.38
*Fannyhessea vaginae*	4/20 (20%)	2/20 (10%)	21.66–36.44	4/19 (21%)	0.09–86.63
Staphylococcus aureus	3/20 (15%)	3/20 (15%)	0.33–16.68	1/19 (5%)	0.04
Staphylococcus saprophyticus	3/20 (15%)	3/20 (15%)	0.79–6.67	ND	ND
Corynebacterium coyleae	3/20 (15%)	3/20 (15%)	0.08–12.70	3/19 (16%)	0.09–1.54
Citrobacter koseri	3/20 (15%)	1/20 (5%)	99.98	3/19 (16%)	0.03–96.62
Actinotignum schaalii	2/20 (10%)	1/20 (5%)	0.28	2/19 (10%)	0.08–0.55
Aerococcus urinae	2/20 (10%)	2/20 (10%)	0.12–3.17	ND	ND
Alloscardovia omnicolens	1/20 (5%)	1/20 (5%)	24.55	ND	ND
Pseudomonas putida	1/20 (5%)	1/20 (5%)	14.72	ND	ND
Stenotrophomonas maltophilia	1/20 (5%)	1/20 (5%)	0.13	ND	ND

a Species are listed in order of decreasing detection frequency in FUM.

b Total detection in FUM of 20 participants by both methodologies. ND, not detected; RA, relative abundance.

## DISCUSSION

Understanding the microbial composition of the lower urinary tract in asymptomatic individuals is essential so that microbial changes associated with urinary disorders can be recognized and modulated as a therapeutic strategy. In this study, using a complementary approach supported by two methodologies (extended culturomics and amplicon sequencing), we expanded the knowledge of the composition of bacterial species within the premenopausal adult female lower urinary tract microbiome.

Each technique presented a different capacity to characterize urinary microbiome profiles (~37% of CSTs overlap for both methodologies), and only 22% of bacterial species were detected by both methodologies. Predictably, amplicon sequencing allowed more frequent detection of slow-growing species (e.g., Campylobacter ureolyticus) and obligate anaerobes (e.g., Finegoldia magna) that require particular culturing conditions. Interestingly, amplicon sequencing also revealed high species diversity within certain anaerobic genera (e.g., *Anaerococcus*, *Peptoniphilus*); however, it is unclear if all these species were viable at the time of detection. On the other hand, the cultured isolates could be accurately identified to the species level, thus providing a higher level of resolution and allowing further investigation to unveil their symbiotic or pathogenic potential. Moreover, some species detected in low-read counts were also identified by extended culturomics, which supports that FUM bacterial diversity reported from DNA-based studies may be underestimated, as also pointed out by other studies (27). Overall, the complementarity of both methodological approaches allowed for a more comprehensive description of the FUM diversity in the studied cohort.

Clustering FUM at the genus level revealed that the most prevalent CST was characterized by the combination of highly abundant *Lactobacillus* and other genera, confirming the previously reported high occurrence of *Lactobacillus* in the female urinary microbiome (3, 10, 13, 17). At the species level the diversity largely increased, with the majority of the CSTs being represented by different *Lactobacillus* or *Gardnerella* species in different RAs and in combination with species from other genera, including low-abundance FUM members, as observed in our previous study (17).

The cooccurrence of *Fannyhessea vaginae* (formerly Atopobium vaginae) and Gardnerella vaginalis has been previously described in the vaginal microbiome, mostly associated with bacterial vaginosis (28, 29). In this study, we identified for the first time a CST dominated by *Fannyhessea vaginae* (RA, 33 to 87%) in combination with *Gardnerella swidsinskii* (RA, ~50%) (Fig. 1, Tables S5 and S6) in the urine of an asymptomatic woman (U15a) (Fig. 2) who did not report any symptoms associated with urogenital diseases. Overall, our findings support that the asymptomatic urinary microbiome might be colonized by *Fannyhessea vaginae*, opportunistic uropathogens (e.g., E. coli, *C. koseri*, or E. faecalis), or species recently associated with urinary disorders (e.g., Aerococcus urinae, Lactobacillus gasseri) (Table 3, Fig. 1) (30, 31). The detection of opportunistic pathogens, in some cases in high RA (e.g., sample U26a) highlights the need to reevaluate the traditional nomenclature initially proposed under the context of the sterile bladder paradigm, such as asymptomatic bacteriuria, long considered a poorly understood phenomenon. In fact, asymptomatic bacteriuria management has evolved toward recommending non-antibiotic treatment in patients without risk factors, in order to avoid the risk of selecting antimicrobial resistance and eradicating a potentially protective bacterial strain (32). Now, a step forward to incorporate these new FUM findings in the clinical guidelines and practice is urgent, but for that, further elucidation of the function of urinary microbiome members, including characterization (presence and expression) of virulence factors *sensu stricto* playing a significant role in pathogenesis, will likely help to understand the development of urogenital diseases (33, 34).

Interestingly, we detected an outstandingly high diversity of species belonging to *Corynebacterium*, *Lactobacillus*, and *Limosilactobacillus* that has not been reported in previous studies characterizing the asymptomatic FUM (Tables S5 and S6) (1–3, 7, 10, 13, 35–37). We also identified 4 *Gardnerella* species in the urinary microbiome of asymptomatic women, according to recent genus reclassification (15). This demonstrates that the high number of colonies studied and reliable identification of isolates by specific genotypic markers, together with the usage of cutting-edge long-read sequencing of the 16S rRNA gene, increase the knowledge on the composition of the bacterial community to the species level in urinary microbiome studies (17, 25, 38).

Additional strengths of this study include sample processing up to 2 h after collection, allowing us to identify anaerobic bacteria that seem to significantly contribute to the urinary microbiome repertoire (27) but are rarely or not reported by other healthy FUM culturomics studies (e.g., Prevotella corporis) (3, 7, 18). Methodological improvements include also the use of a larger volume sample size (20 mL) than the previously used urine volume (mostly 1 mL) in DNA extraction protocols, which increased the high-quality microbial DNA yield required for high-resolution sequencing and unveiled detection of species not previously reported in DNA-based studies (e.g., Alistipes putredinis) (1, 10, 12, 27). Another important improvement was the use of a cutting-edge sequencing technique, including near full-length 16S rRNA gene sequencing using PacBio SMRT cell technology (25, 39–41), and appropriate gene markers to identify cultured isolates at the species level, which enabled increased taxonomic resolution, as well as validation of several low-read sequencing data (<0.1% RA) by our extended culturomic protocol.

This study presented some limitations. Urinary microbiome CSTs identified in this work should be validated on a larger cohort, including participants representing a homogeneous asymptomatic female group (e.g., no antibiotics for any medical reason within the month prior to urine collection and samples collected in 3rd week of menstrual cycle). Although the selected participants had not been on antibiotics in the previous month, it cannot be excluded that effects of antibiotics can last longer than a month and even lead to persistent changes in the urinary microbiome, as previously observed in the gut microbiome (42). The focus on the urinary bacterial community within the healthy FUM overshadows the potential role of nonbacterial components (e.g., fungi, viruses, and archaea) contributing to a healthy urinary microbiome. Importantly, the small sample size used in this study could increase a risk of CSTs overfitting, especially in culturomic species-level analyses. The high sensitivity of culturomics to differentiate closely related species and detect low-abundance members is likely to generate complex data sets that can constitute a challenge for clustering analysis. Although suitable for genus-level data and amplicon sequencing of species-level data, the cutoff of 0.8 used for cluster delineation may not reflect reliable microbiome patterns in a complex culturomic species-level data set, especially when sample size is not sufficient to provide statistical power.

Additionally, culturomics is prone to provide a biased estimation of bacterial counts (e.g., highly similar morphological appearance, growth requirements promoting certain bacteria), and we possibly underestimated the diversity of the urinary microbiota, even with the meticulous protocol implemented. The two methodologies used in this study are substantially different in their capacity to capture bacterial species. Despite culturomics representing enhanced culturing that had been greatly improved over past years to grow fastidious bacteria, it is unquestionable that sequencing-based analysis provides more robust microbiome profiling. These differences in sampling depth likely contributed to moderate correlations between the urinary microbiome profiles obtained by the two methodologies.

The use of voided urine instead of urine collected by suprapubic aspiration or urethral catheterization could be also considered a limitation (43, 44). However, suprapubic aspiration or catheterization of participants who were not at a high risk of bacterial infection or without any clinical urinary symptoms was not ethically feasible as per our local ethics committee. Moreover, voided urine is a sample commonly used for diagnosis of urinary tract pathologies and captures the urethral bacteria, which can play an important role in urinary tract conditions.

**Conclusions.** Our study substantially increased the knowledge of bacterial species diversity in the FUM of reproductive-age asymptomatic women and provided extensive taxonomic characterization of the genera *Gardnerella* and *Corynebacterium* and the family *Lactobacillaceae*, which are prevalent members in this niche. We demonstrated that, at the species level, FUM is highly diverse within and between individuals, and the most prevalent FUM members are low-abundance bacteria, potentially playing an important role in urinary tract eubiosis.

This study provides a fine-grained analysis using culture- and DNA-based approaches to capture FUM species-level diversity. Additionally, the data provided here can be useful to estimate the bias resulting from using just one methodology.

Finally, our findings provide essential species-level information for further studies of microbiome dysbiosis associated with urinary tract infection and lower urinary tract symptoms, which is required for development of more effective diagnostic and/or therapeutic strategies. As we begin to detect near-full composition and diversity of the urinary microbiome, future studies on functional properties of the resident microbiome in the human urinary tract should receive high priority.

## MATERIALS AND METHODS

### Participants and sample collection.

This study was approved by the Faculty of Pharmacy (University of Porto, Porto, Portugal) Ethics Committee, and written informed consent was obtained from all study participants. A total of 20 women of reproductive age were recruited between November 2016 and July 2018, following strict criteria: no pregnancy, no symptoms or diagnosis of current UTI, and no antibiotic exposure in the previous month. A questionnaire was conducted concerning personal and health information that was encrypted, ensuring data confidentiality. Participants were carefully instructed in the collection technique. Considering physiological changes ongoing within the female genital tract during the menstrual cycle and possibility that it could influence the composition of the urinary microbiome, we chose to collect samples always in the same phase of the menstrual cycle. Since most drastic changes are observed during menstruation (e.g., bloody discharge, increased pH, and lower estrogen levels), we considered the third week to be distant enough for the vaginal microbiome to stabilize its composition. Consequently, in the third week of the menstrual cycle, each participant provided a first-morning midstream voided urine sample by a self-performed noninvasive procedure via 40-mL sterile containers.

Urinary dipstick (Combur-Test, Roche) analysis and microscopic examination of the resuspended sediment of centrifuged urine (1 mL) were performed. Up to 2 h after collection, urine samples were subjected to an extended culturomics protocol, concurrently pretreated for amplicon sequencing analysis, and stored at −80°C. The FUM culturomics data from 10 women, published in the context of urinary tract microbiome temporal stability (17), were included in this study. Since this manuscript includes novel data from amplicon sequencing performed on the same samples, previous culturomics data were used for comparison of efficacy of two methodologies and accurate assessment of community structure types.

### Extended culturomics.

The extended culturomics protocol included inoculation of 0.1 mL of urine onto a large plate surface (140-mm diameter) of Columbia agar with 5% sheep blood (blood agar plates [BAPs], Biogerm, Portugal) and HiCrome UTI agar (chromogenic agar plates [CAPs], HiMedia, India) supplemented as previously described (45, 46). BAPs and CAPs were incubated under aerobic and microaerophilic conditions (GENbox Microaer, bioMérieux) at 37°C for 48 h. Additionally, BAPs were incubated under anaerobic conditions (GENbox Anaer, bioMérieux) at 37°C for 48 h. In the case of a suspected high bacterial load based on microscopic observation, 10-fold serial dilutions (up to 0.001) were performed using sterile saline solution (0.9% NaCl) to obtain a countable range of CFU/mL. Each morphologically distinct colony type was counted, and 1 to 5 colonies of each morphology were further identified. The plate presenting the higher CFU count was considered the representative count of each isolate in a sample. When more than one species was identified within the same colony morphotype, the CFU count was split proportionally between the identified species. Relative abundance (RA [%]) was calculated by generating the percentage of total CFU/sample.

### Identification of cultured bacteria.

MALDI-TOF MS with the *in vitro* diagnostic (IVD) database version 3.0 (Vitek MS automation control and Myla software, bioMérieux, France) was used to identify the bacterial isolates. Isolates with no identification, with discrepant results between MALDI-TOF MS identification and phenotypic characteristics, or with known insufficient resolution power for species identification were further subjected to sequencing of the 16S rRNA gene and/or other genetic markers (*pheS* for *Lactobacillus* and *Limosilactobacillus*, *cpn60* for *Gardnerella*, *rpoB* for Acinetobacter, *Corynebacterium*, or Staphylococcus, and *recN* for *Citrobacter*) and/or PCR assays for the detection of species-specific genes (*dltS* for group B Streptococcus, *sodA* for Enterococcus faecalis, and *malB* for Escherichia coli) (Table S1). GenBank accession numbers and species identification for FUM isolates subjected to Sanger sequencing are available in Table S2 and were previously published by Ksiezarek et al. (17). Phylogenetic analysis based on individual genes was performed to access putative novel species by using MEGA version 7.0 (47), constructed according to the neighbor-joining method (48), and genetic distances were estimated using Kimura’s 2-parameter model (49). The reliability of internal branches was assessed from bootstrapping based on 1,000 resamplings (50).

### DNA extraction and amplicon sequencing.

Samples were pretreated prior to DNA extraction, which included centrifugation of 20 mL of urine at 5,500 rpm for 15 min, and the resulting pellet was suspended in 1 mL of phosphate-buffered saline (PBS) and stored at −80°C until further processing. PBS was discarded by centrifugation at 10,500 rpm/15 min/4°C immediately before genomic extraction. Genomic DNA from urine samples was extracted using the Qiagen DNeasy blood and tissue kit (Qiagen, Germany) according to the manufacturer’s protocol, using pretreatment for Gram-negative bacteria (51). DNA was eluted into 50 μL of Tris-HCl (pH 8.0) and stored at 4°C. DNA quality was analyzed by agarose gel electrophoresis, and quantity was measured on a Qubit double-stranded DNA (dsDNA) high-sensitivity (HS) assay kit (Invitrogen, Life Technologies, UK). Negative controls consisting of reagent blanks (washing buffer, lysis buffer, and kit reagents) were processed the same way as the urine samples. Since extraction controls showed no traceable amounts of DNA, they were not included for sequencing. PCR amplification of the hypervariable 16S rRNA gene V1 to V8 regions sequenced with universal primers (27F: AGAGTTTGATCCTGGCTCAG and BS-R1407: GACGGGCGGTGWGTRC), library construction, and sequencing with SMRT technology on a PacBio RS II sequencing system were provided as a custom service of Eurofins GATC Biotech GmbH (Germany). DNA from negative controls was sent to Eurofins GATC Biotech GmbH, yet the minimal concentration of DNA required to be amplified was not met and thus could not be sequenced by the company. Nevertheless, we performed the amplification of DNA from negative controls by nested PCR (1st PCR: primers F27 and R1492 [52], 2nd PCR: for V3/V4 hypervariable region of the 16S rRNA gene [53, 54]). No amplification products were observed; indeed, the agarose gel showed no amplification bands, and the DNA quantification with the Qubit kit provided no detectable DNA in the negative control. Negative and positive (mock community) controls were included by the company doing the sequencing to ensure that no large-scale cross-contamination between samples took place and that the sequencing itself did not introduce any errors.

### Sequencing data analysis.

After sequencing, primers, sequence adaptors, and low-base-quality calls were removed using Cutadapt. Chimera sequences were checked and removed using UCHIME (version 4.2.40) (55). The nonchimera and unique sequences were subjected to BLASTn (56) analysis using nonredundant 16S rRNA reference sequences with an E-value cutoff of 1e-06. Reference 16S rRNA gene sequences were obtained from the Ribosomal Database Project Classifier (57). Only good-quality and unique 16S rRNA sequences which have a taxonomic assignment were considered and used as a reference database to assign operational taxonomic unit (OTU) status with a 97% similarity. Taxonomic classification was based on NCBI Taxonomy (58). All the hits to the reference 16S rRNA database were considered, and specific filters were applied to the hits to remove false positives. The thresholds applied were ≥97.00% identity, ≥95.00% alignment coverage, 1,000 minimum query length, 10% bitscore threshold for multiple hits, and 250 maximum hits to consider for multiple hits. If the final number of high-quality reads after all filtering steps was less than 1,000, the corresponding sample was excluded. Finally, RA was calculated by generating the percentage of total reads for each sample.

### Statistical analysis.

Community structural analyses were performed based on the RAs of genera and species within each sample. Based on the similarity (or dissimilarity) of community composition between samples and taking into account all members and their RAs in a community, we identified community structure types performing hierarchical clustering of Bray-Curtis dissimilarity distance matrices with a cutoff of 0.8 via the vegan package (version 2.5-2) (59) in R (version 3.4.4) (60). The dissimilarity cutoff of 0.8 used when performing the cluster analysis indicates that the samples were clustered at ≥80% similarity, considering both species presence/absence and their RA. Alpha diversity was estimated using the Shannon index (H′). Hierarchical clustering of Bray-Curtis dissimilarity distance matrices by both methodologies, principal-coordinate analysis (PCoA), and Mantel test between the dissimilarity distance matrices (based on the Bray-Curtis index) were performed to compare structure types obtained by both methodologies. To identify the species responsible for community structure differences, a biplot of the PCoA was created using a weighted average of the species scores based on their RA in the samples. Data visualization was carried out using the gplots (version 3.0.1.1) (61), ggplot2 (version 3.2.1) (62), and eulerr (version 5.1.0) (63) R packages.

### Ethics approval and consent to participate.

Approval of the study was obtained from the Faculty of Pharmacy (University of Porto, Porto, Portugal) Ethics Committee. Procedures performed in the study were all in accordance with the ethical standards of the institutional and national research committee and with the 1964 Helsinki Declaration and its later amendments. All individual participants included in the study have written informed consent.

### Data and materials availability.

The data sets supporting the conclusions of this article are included within the article and its supplemental material and available in the Sequence Read Archive repository, under BioProject accession number PRJNA548360.
