# Supplementary material for: Urinary Microbiome of Reproductive-Age Asymptomatic European Women
Source: Microbiol Spectr. 2022 Nov 16;10(6):e01308-22. doi: 10.1128/spectrum.01308-22 (PMC9769847; doi:10.1128/spectrum.01308-22)
Supplement: Supplemental file 1 — Fig. S1 to S5. Download spectrum.01308-22-s0001.pdf, PDF file, 0.4 MB [file spectrum.01308-22-s0001.pdf]

Supplementary Figures S1-S5.

Urinary microbiome of reproductive-age healthy European women

Svetlana Ugarcina Perovic, Magdalena Ksiezarek, Joana Rocha, Elisabete Alves Cappelli,

Márcia Sousa, Teresa Gonçalves Ribeiro, Filipa Grosso, Luisa Peixe

A

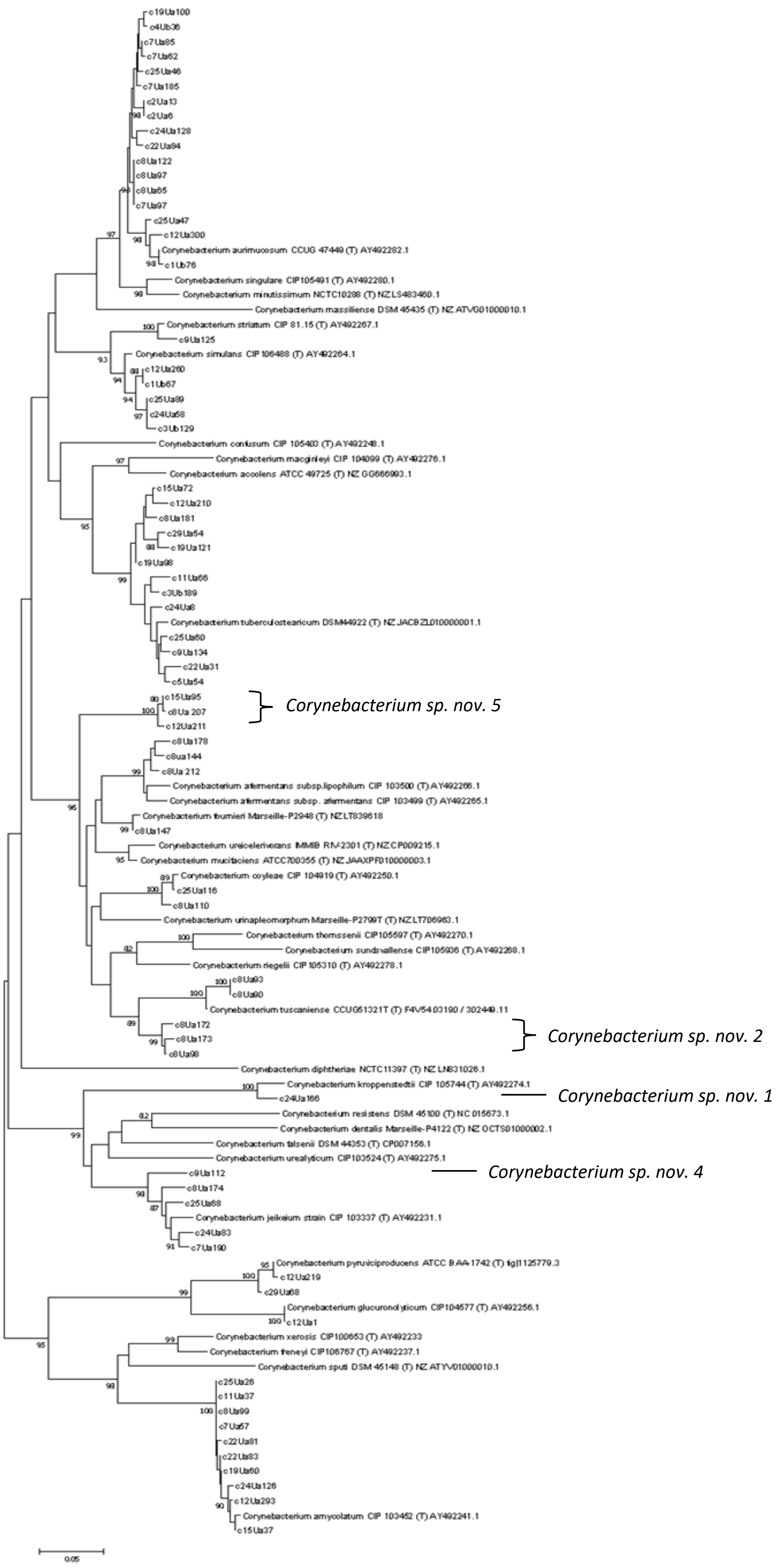
Corynebacterium sp. nov. 5
Corynebacterium sp. nov. 2
Corynebacterium sp. nov. 1
Corynebacterium sp. nov. 4
0.05

B

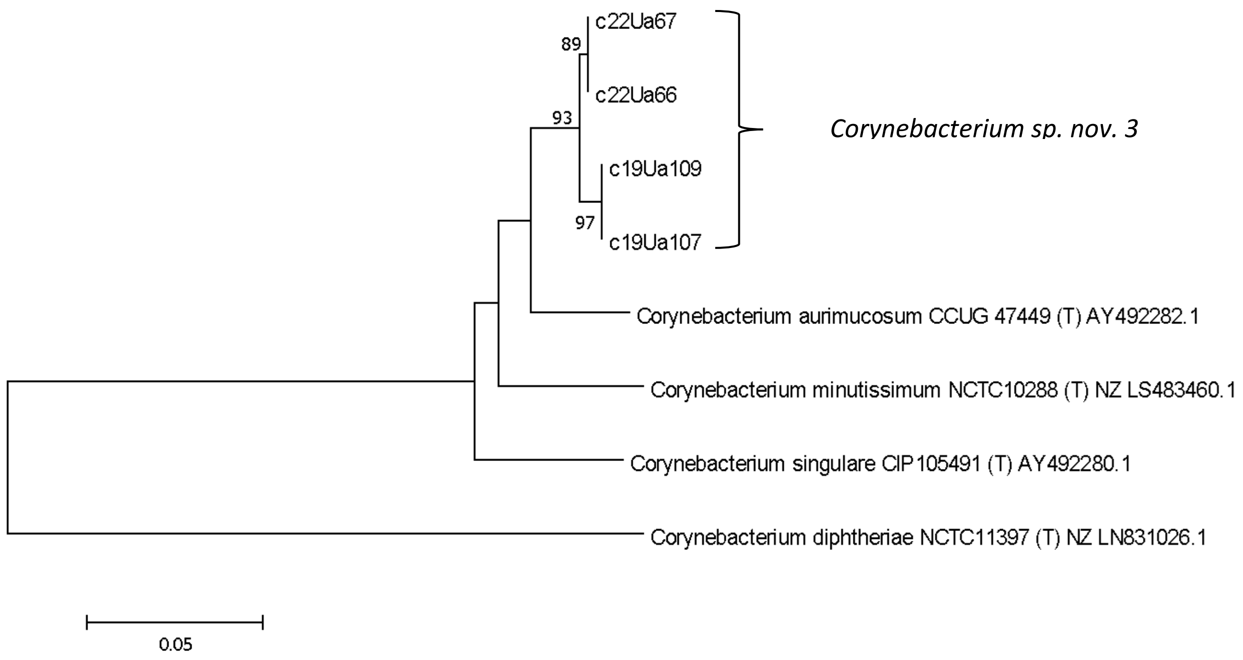

Figure S1. *Corynebacterium* putative novel species 1-5. Neighbor-joining tree based on *rpoB* gene sequences showing the phylogenetic relationships between *Corynebacterium* selected closely related type strains and putative novel species. Nucleotide sequences were extracted from draft/complete genomes obtained from the NCBI Assembly Database, for which the accession numbers are shown next to the strain designation. Bootstrap percentages (based on 1000 replications) are shown at nodes. Only values above 80% are shown. Bar, 0.05 substitutions per nucleotide position. Figure S1A represents isolates sequenced with primers F- CGWATGAACATYGGBCAGGT and R- TCCATYTCRCCRAARCGCTG with ~450 bp amplicon, while figure S1B includes isolated sequenced with primers F-CNTCBCACTAYGGNCGNATG and R- GAVCGNGCGTGRATCTTYTC with ~1700 bp amplicon.

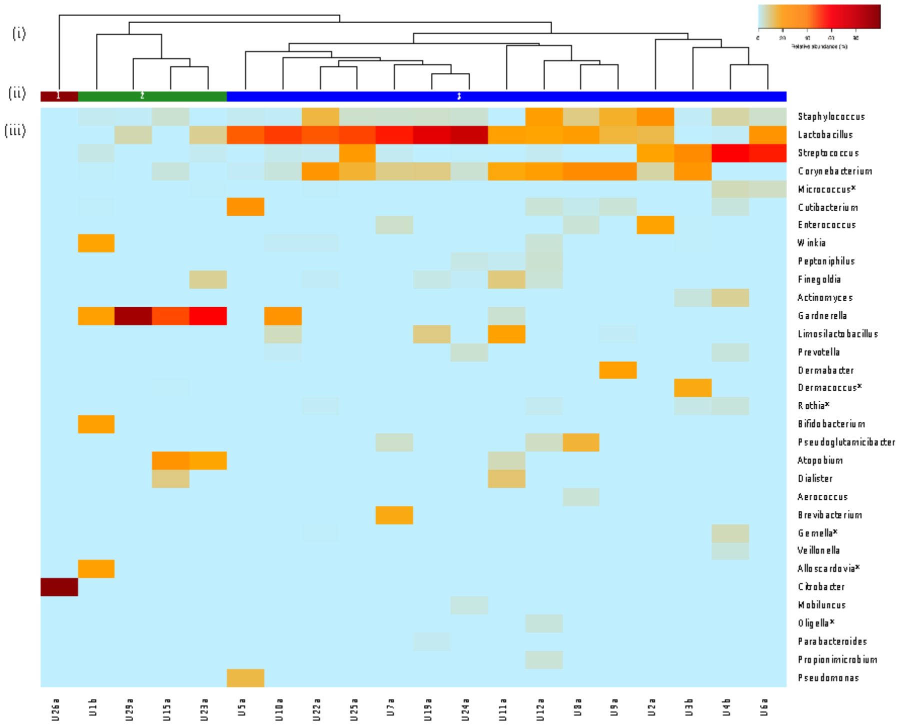

Figure S2. Genus-level community structure types of healthy FUM by culturomics.

(i) Hierarchical clustering of Bray-Curtis dissimilarity distance matrices on the relative proportions of CFU/ml within individual urine samples. (ii) Bars below dendrogram denote community structure types. (iii) Heatmap of relative abundances of bacterial genera within each urinary microbiota. Only genera that are at least 1% abundant in at least one sample are shown in order of decreasing prevalence (from top to bottom). Asterisk (*) denotes detection only by culturomics and not by community amplicon sequencing.

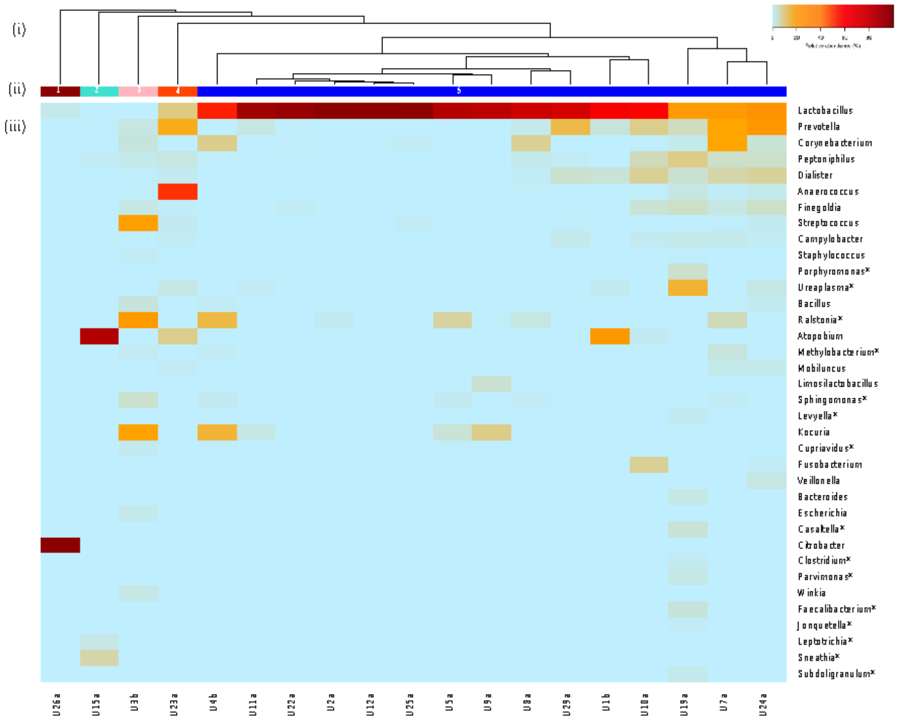

Figure S3. Genus-level community structure types of healthy FUM by community amplicon sequencing.

(i) Hierarchical clustering of Bray-Curtis dissimilarity distance matrices on the relative proportions of reads for each OTU within individual urine samples. (ii) Bars below dendrogram denote community structure types. (iii) Heatmap of relative abundances of bacterial genera within each urinary microbiota. Only genera that are at least 1% abundant in at least one sample are shown in order of decreasing prevalence (from top to bottom). Asterisk denotes detection only by community amplicon sequencing and not by culturomics.

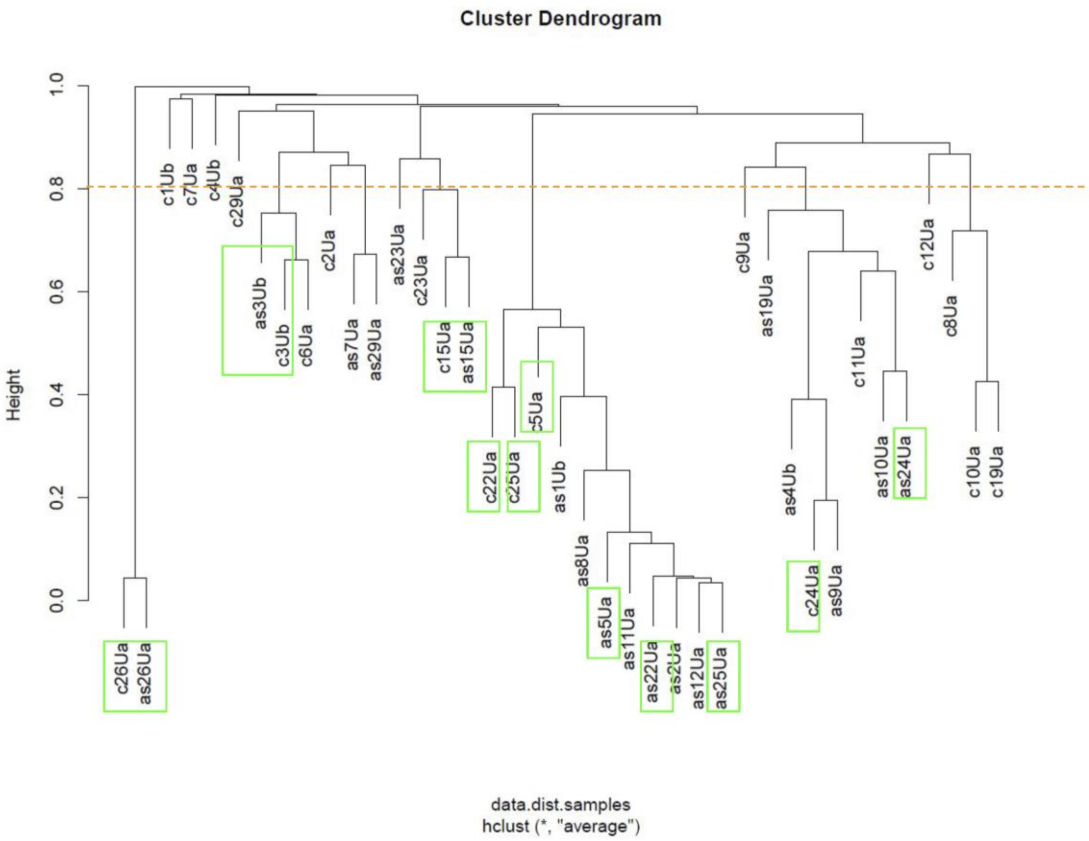

Figure S4. Species-level hierarchical clustering including 19 samples characterized by culturomics (c) and amplicon sequencing (as). Hierarchical clustering was based on Bray-Curtis dissimilarity distance matrices, for which all species detected were included. A cutoff value of 0.8 was used to define the clusters (dashed orange line). Samples clustered into the same CST by both methodologies are shown in green boxes.

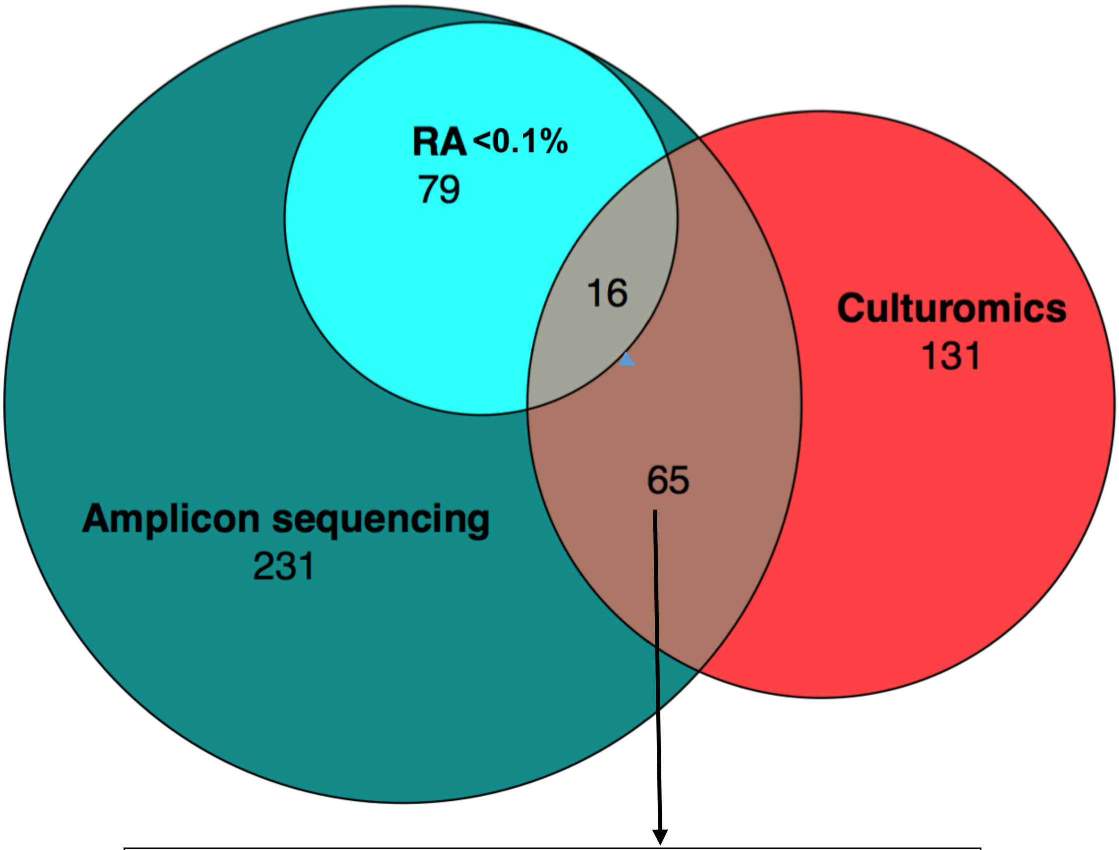

Species detected by both methodologies		
*Abiotrophia defectiva**<br>*Acinetobacter* spp.*<br>*Actinomyces europaeus*<br>*Actinomyces* spp.*<br>*Actinomyces urogenitalis**<br>*Actinomyces viscosus**<br>*Actinotignum schaalii*<br>*Anaerococcus tetradius*<br>*Atopobium minutum*<br>*Bacillus simplex**<br>*Bacillus* spp.<br>*Bacteroides vulgatus*<br>*Bifidobacterium* spp.*<br>*Campylobacter ureolyticus*<br>*Citrobacter koseri*<br>*Collinsella aerofaciens*<br>*Corynebacterium aurimucosum*<br>*Corynebacterium coyleae*<br>*Corynebacterium jeikeium*<br>*Corynebacterium riegelii*<br>*Corynebacterium simulans*	*Corynebacterium tuberculostearicum*<br>*Cutibacterium acnes*<br>*Dermabacter* spp.*<br>*Dialister micraerophilus*<br>*Dialister propionicifaciens*<br>*Enterococcus faecalis*<br>*Escherichia coli*<br>*Facklamia hominis*<br>*Fannyhessea vaginae*<br>*Finegoldia magna*<br>*Fusobacterium nucleatum*<br>*Granulicatella adiacens**<br>*Lactobacillus crispatus*<br>*Lactobacillus delbrueckii*<br>*Lactobacillus gasseri*<br>*Lactobacillus iners*<br>*Lactobacillus jensenii*<br>*Limosilactobacillus mucosae*<br>*Limosilactobacillus vaginalis*<br>*Mobiluncus curtisii*<br>*Moraxella* spp.*<br>*Peptoniphilus asaccharolyticus*	*Peptoniphilus coxii*<br>*Peptoniphilus duerdenii*<br>*Peptoniphilus lacrimalis*<br>*Prevotella bivia*<br>*Prevotella corporis*<br>*Prevotella disiens*<br>*Propionimicrobium lymphophilum*<br>*Pseudoclavibacter* spp.*<br>*Rhizobium radiobacter**<br>*Slackia exigua**<br>*Staphylococcus aureus**<br>*Staphylococcus epidermidis*<br>*Staphylococcus haemolyticus*<br>*Staphylococcus hominis*<br>*Staphylococcus lugdunensis**<br>*Staphylococcus* spp.<br>*Streptococcus agalactiae*<br>*Streptococcus anginosus*<br>*Streptococcus gordonii**<br>*Streptococcus* spp.<br>*Veillonella parvula*<br>*Winkia neuii*

* species detected by amplicon sequencing in relative abundance < 0.1%

Figure S5. Venn-Euler diagram showing the number of species (N = 297) detected by culturomics and/or amplicon sequencing. The size of the circles and intersections is proportional to the number of species detected. Species detected by both methodologies are listed in alphabetical order. (RA, relative abundance)
